# Examining Honeybee (*Apis mellifera*) Dominance Patterns Within Urban Bee Communities Worldwide

**DOI:** 10.1002/ece3.71979

**Published:** 2025-08-13

**Authors:** Joan Casanelles‐Abella, Julieta Badini, Katherine Baldock, Ana Calviño, Maria Silvina Fenoglio, Sara Diana Leonhardt, Astrid Neumann, Marco Moretti, Mark Patterson, Bruno Rossi‐Rotondi, Aaron Sexton, Karla Palmieri Tavares, Juan Pablo Torretta, Martin Videla, Fernando Zamudio, Raffael Zenni, Monika Egerer

**Affiliations:** ^1^ Urban Productive Ecosystems, TUM School of Life Sciences Technical University of Munich Freising Germany; ^2^ Biodiversity and Conservation Biology Swiss Federal Research Institute WSL Birmensdorf Switzerland; ^3^ Instituto Multidisciplinario de Biología Vegetal (IMBIV) Universidad Nacional de Córdoba (UNC)—CONICET Córdoba Argentina; ^4^ Department of Geography and Environmental Sciences Northumbria University Newcastle upon Tyne UK; ^5^ Plant‐Insect Interactions, TUM School of Life Sciences Technical University of Munich Freising Germany; ^6^ Api:Cultural London UK; ^7^ Programa de Pós‐Graduação Em Ecologia Aplicada, Instituto de Ciências Naturais Universidade Federal de Lavras Lavras Brazil; ^8^ Federal Institute of Southern Minas Gerais—Machado Campus Machado Brazil; ^9^ Facultad de Agronomía, Cátedra de Botánica General Universidad de Buenos Aires, CONICET Buenos Aires Argentina

**Keywords:** Anthophila, bees, diversity distribution, urban beekeeping, urban ecosystems, Western European honeybee

## Abstract

Urban ecosystems can host diverse bee communities. However, the increasing prevalence of urban honeybees ( *Apis mellifera* Linnaeus 1758) raises concerns about their ecological impact. Using a systematic review of published studies, we obtained 68 datasets representing 46 cities in 15 countries and five continents to test the extent to which honeybees are dominant in urban bee communities worldwide. Honeybees ranked as the most abundant species in ca. 70% of the datasets and accounted for more than 10% of all individuals in ca. two‐thirds of the datasets. Moreover, honeybees ranked among the top three abundant species in 70% of studies. Honeybee abundance patterns were consistent across regions and sampling designs, independent of whether honeybees were native or not. At the same time, the degree of dominance varied across cities. These findings highlight the need to address the ecological implications of honeybee dominance, including assessing the effects on wild bee communities and populations and defining strategies to enhance, preserve wild bees, and enhance coexistence with honeybees.

## Graphical abstract

We examine if and to what degree honeybees are dominant in urban bee communities worldwide. We found honeybees to be the most abundant organisms and highly dominant in most of the studies, although the dominance degree was quite variable between studies. Our results provide new evidence to contribute to the debate on urban beekeeping and reconciliation with wild pollinator conservation.

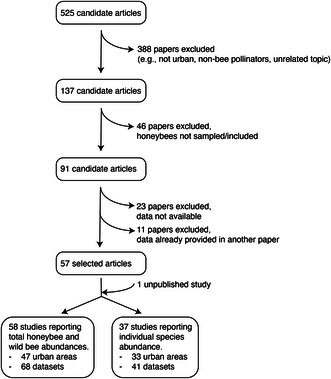

## 1 Introduction

Urban ecosystems can harbor a relatively high diversity of bees compared with other anthropogenic ecosystems such as highly intensified agricultural areas. High flowering plant diversity, an abundance of nesting structures, especially above‐ground cavities, and lower concentrations of pesticides and herbicides are important factors for supporting diverse urban bee communities (Baldock et al. 2019; Brom et al. 2022). High bee diversities have been found across the world, including cities in Africa (e.g., Guenat et al. 2019), Europe (e.g., Baldock et al. 2019; Kratschmer et al. 2018), North America (Anderson et al. 2023; Lowenstein et al. 2015), and Australia (e.g., Persson et al. 2022; Prendergast et al. 2022). Moreover, over the last two decades, multiple cities have actively engaged in supporting bees and other pollinators through changes in management regimes of urban green spaces (e.g., mowing relaxation Chollet et al. 2018; Lerman et al. 2018) or restoration programs which support or expand favorable pollinator habitat across urban green spaces (Baldock 2020).

Western honeybee ( *Apis mellifera* Linnaeus 1758; honeybees hereinafter) populations are found in multiple cities across all continents due to potentially two related processes. The first is the rising popularity of urban beekeeping (i.e., beekeeping within cities), which is a relatively new phenomenon for most cities (Egerer and Kowarik 2020). This process has increased the number of managed honeybee individuals kept within cities (Casanelles‐Abella and Moretti 2022; MacInnis et al. 2023). The second is due to beekeeping occurring within the surrounding areas of cities (i.e., beekeeping outside of cities and in the peri‐urban fringe). In such cases, honeybees can spill over to urban areas to temporarily forage on urban floral resources (Samuelson et al. 2022) and might also establish feral colonies (Rotondi et al. 2023). Large increases in honeybee populations placed in landscapes have raised alarms regarding the potential ecological consequences on wild bees and other pollinating insects; scientists have hypothesized that with increasing honeybee densities and resource overlap, there will be an increased risk of competition for floral resources (Ropars et al. 2019; Sponsler and Bratman 2021), modulated by both the feeding niche partitioning traits of the bees and the plant pollination‐related traits (Cappellari et al. 2022; Casanelles‐Abella et al. 2023; Page et al. 2024) and altered or enhanced disease transmission (Maurer et al. 2024). The consequences of increased competition and disease transmission are complex and might have a temporal gap before they become apparent (Sponsler et al. 2023; Sponsler and Bratman 2021). Nonetheless, prior research has documented reduced community abundance and diversity (Valido et al. 2014), reduced populations of targeted bees (MacKell et al. 2023), impoverishment of bee diet quality (Page et al. 2024; Page and Williams 2023), and altered foraging activity time (Pasquali et al. 2025).

Investigating the effects of increased honeybee populations is important not only for understanding the factors shaping urban bee diversity broadly (Kilpinen et al. 2022) but also provides the basis for informing management recommendations and policy frameworks (Beaurepaire et al. 2025). A number of studies have investigated the potential negative impact of urban honeybees on wild bees, but this research remains limited to a few cities and kinds of study sites (e.g., parks, gardens) including Paris, France (Ropars et al. 2019), Munich, Germany (Renner et al. 2021), Zurich, Switzerland (Casanelles‐Abella et al. 2023), Vienna, Austria (Lanner et al. 2025), Montréal, Canada (MacInnis et al. 2023; McCune et al. 2020) Cordoba, Argentina (Rotondi et al. 2023) and several Brazilian cities (Tavares Brancher et al. 2024). This is unfortunate because negative consequences of urban beekeeping are expected to be heightened in cities located outside of the natural distribution ranges of honeybees (outside Europe, Africa and Western Asia) due to potential invasion (Ackerman 2021; Moritz et al. 2005), as seen for both honeybees (Iwasaki and Hogendoorn 2022) and other commercially used bee species such as *Bombus terrestris* (Linnaeus, 1758) (Morales et al. 2013). In this regard, honeybees are recognized as a particularly successful invasive species because they can rapidly increase their population in their range, they are often cared for and fed by beekeepers and, as generalists, exploit a wide range of floral resources in the landscape.

To understand the impacts of honeybees on wild bees worldwide, it is important to assess the distribution of abundances and dominance patterns of urban bee communities, including both wild bees and managed honeybees. The distribution of abundance and dominance patterns can inform how ecological processes such as niche partitioning or community stability occur (Brown 1984; Ulrich et al. 2010). Moreover, as stated by Kilpinen et al. (2022) “*dominance of one bee species is a marker for bee community degradation, and often but not always the superorganism honeybee colony is the dominant bee*”, although the dominance of other bee species also leads to negative impacts on bee communities (Garibaldi et al. 2021).

As social organisms linked to human facilitation, one may predict that urban honeybees are highly abundant and consequently very dominant through their nature. However, prior work in other ecosystems has shown that the extent or degree to which honeybees dominate in a community (e.g., the contribution of honeybee individuals to the overall bee community) might be quite variable (Garibaldi et al. 2021; also discussed in Kilpinen et al. 2022). Cities have different intensities of urban beekeeping as well as densities of feral honeybee populations, both factors that influence honeybee dominance patterns. While some studies have reported a large dominance of honeybees (20%–50% of the total collected individuals; MacInnis et al. 2023; Rotondi et al. 2023; Tavares Brancher et al. 2024), others have reported much lower degrees of dominance (e.g., < 10% of the total collected individuals, Felderhoff et al. 2023; Simao et al. 2018). These contrasting patterns make it difficult to make generalizations of how dominant honeybees are or how variable their degree of dominance is within urban bee communities worldwide. Other factors that can also influence recorded honeybee dominance patterns include the sampling methods (Prendergast et al. 2020), focal sampling habitat, or whether honeybees are native or non‐native in the studied urban area (Garibaldi et al. 2021). Thus, the extent to which urban honeybees are dominant in relation to the remaining urban bee community is a critical question for understanding and assessing the status of urban bee communities.

Here, we investigated dominance patterns of honeybees in urban bee faunas across cities worldwide. We asked the following three questions: (1) how dominant are honeybees relative to wild bees within urban bee communities? (2) does dominance vary depending on sampling and study design? and (3) does the degree of honeybee dominance vary across cities worldwide? To do so, we used 68 datasets from 58 urban bee community studies compiled using a systematic literature review. These data included 15 cities and five continents, representing contexts within and outside honeybee natural distributional ranges (i.e., within and outside Europe and Africa, and Western parts of Asia) and sampling methods (e.g., active (hand‐netting), passive (traps) and a combination of both).

## 2 Methods

To identify potential datasets, we conducted a systematic literature review of the published urban literature using the Scopus database (www.scopus.com) together with the software Publish or Perish (https://harzing.com/resources/publish‐or‐perish), following Hahs et al. (2023). We searched for research papers about “urban” and “bees” with the following key terms: TOPIC (“bee*” OR “pollinator*”) AND (“urban” OR “peri‐urban” OR “periurban*” OR “suburban*” OR “sub‐urban*” OR “conurbation” OR “city” OR “cities” OR “town*” OR “megalopol*” OR “metropol*” OR “built‐up” OR “built environm*”) in the abstract, title, or keywords published from 1990 (following Hahs et al. 2023) up to and including October 2024, in English, in international academic journals.

**FIGURE 1:**
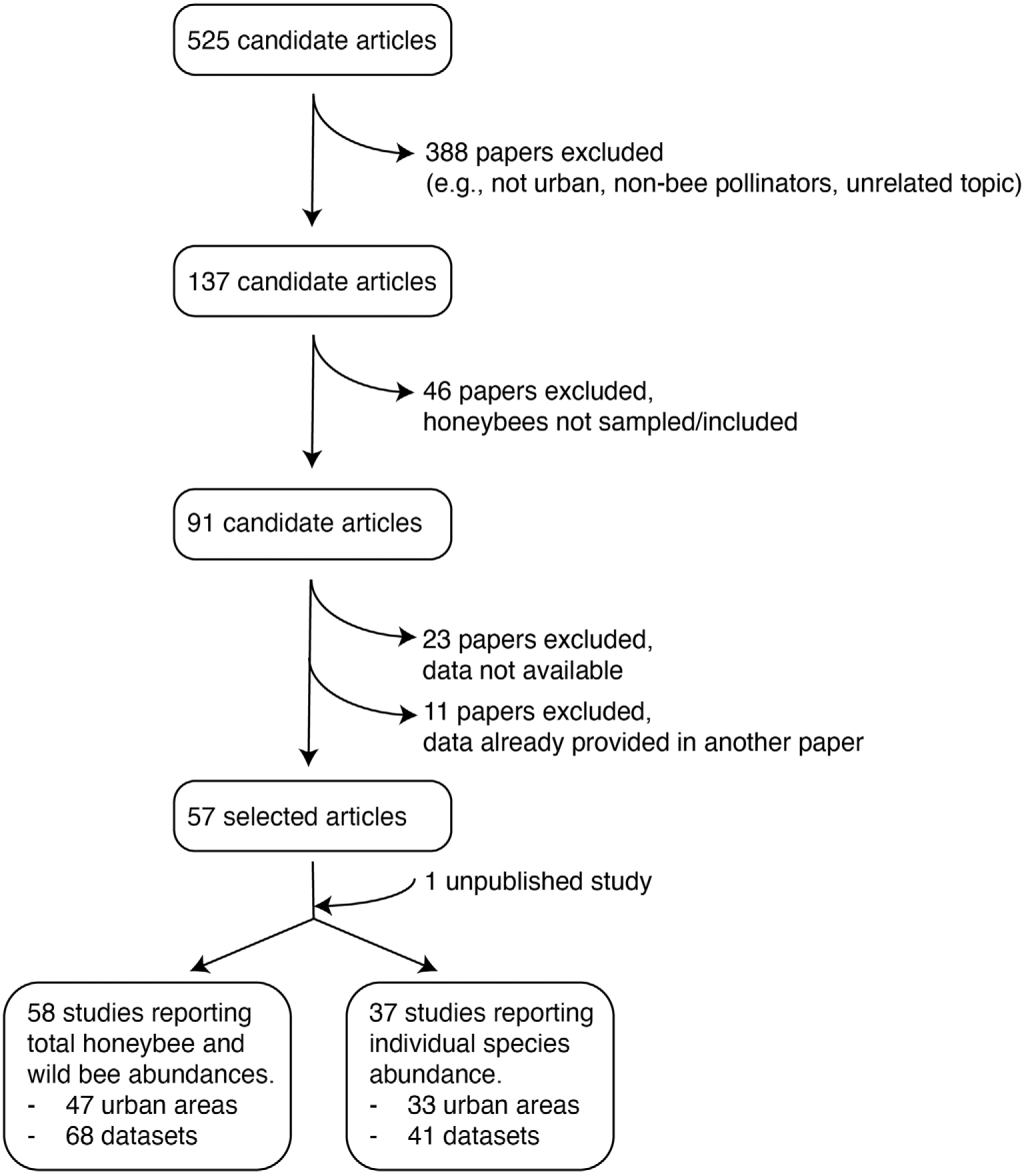
Summary of the literature review and included articles (studies), urban areas and datasets.

The initial search resulted in 525 candidate papers for screening (Figure 1, Data S1). We included only those studies that met the following criteria: (1) studies including community‐level bee data of both honeybees and wild bees, (2) studies reporting abundances within the manuscript, appendix, or in a publicly accessible repository, (3) studies in which sampling took place in one or multiple cities (i.e., excluding those that focused solely on non‐urban ecosystems), and (4) studies where information on the sampling details was available (e.g., type of sampling, duration of the sampling, number of study sites). We looked for studies reporting the total abundances of wild bees and honeybees. Furthermore, when available, we also looked for individual species abundances for investigating the community rank‐abundance distribution. Other article types, such as reviews and perspectives, were not considered.

First, all abstracts, methods and results were screened, and non‐suitable articles were excluded; a total of 388 papers were excluded, resulting in 137 papers (full details can be found in the archived dataset “Results literature review” in Data S1). Among other reasons for exclusion were: focus on non‐bee pollinators, focus on unrelated topics (e.g., traffic models, pollution, nutritional analyses, natural history of specific species, plant focus), not conducted in urban areas, no community data, insufficient taxonomic resolution (e.g., bees identified to guild, family or order level), insufficient taxonomic breadth (i.e., only a subset of the bee community is sampled, such as bumblebees or solitary bees). From the resulting 137 papers, we removed 46 papers (Figure 1 and Data S1) that excluded honeybees from the sampling. Then, for the 91 remaining papers (Figure 1 and Data S1), we screened methods, results, appendices and, when available, data availability statements. After that, we excluded 23 papers (Figure 1) where abundance data was not available within the main manuscript, appendix or from an external, publicly accessible repository, resulting in 68 papers. Moreover, we excluded 11 papers that used the same dataset as another selected article. This resulted in 57 remaining studies. Additionally, we added the data from an unpublished study in Cordoba, Argentina (Badini, Zamudio, Torretta, Calvino, in preparation) that provided data on the proportions of honeybees and wild bees, as well as on the individual bee species abundances.

For 54 out of the 58 studies collected, we could associate the bee community data to a specific city (e.g., Berlin, Zurich, Cordoba). In four studies that sampled bee communities in more than one city, the data were aggregated (Text S1), and it was not possible to obtain city‐specific estimates.

For all 58 studies (57 published studies and 1 unpublished study), we obtained the total wild bee abundance and honeybee abundance for calculating the proportions of the two groups (Figure 1, Data S1). Moreover, for 37 studies (36 published studies and one unpublished study), we also obtained individual abundances for each species to perform the rank‐abundance distribution (Figure 1, Data S1).

We extracted data on the details and content of the studies, including factors that might influence the abundance of honeybees (e.g., sampling type, sampling duration, number of study sites). Specifically, we collected the following information:

- Article basic information: title, authors, doi, web link, year of publication, journal, and publisher.
- Study area: continent, country, number of cities, and city names where the study had been conducted.
- Sampling design: sampling type (i.e., active, passive and both), sampling methods (specifying the method use within sampling type, e.g., pan‐traps, interception traps and equivalent for passive types; observation and hand‐netting within transects for active types), years of the beginning and end of the sampling period, number of sampling years, number of sites within a city, and the city area (in km^2^). We also recorded the habitat/urban greenspace types where bees were sampled, adapting the typology from Aronson et al. (2017) (see also Table S1) and the total number of habitats. For studies sampling more than one habitat/urban greenspace type, as the data was aggregated at the city level, we could not distinguish among individual types.
- Abundance data: honeybee abundance, wild bee abundance, and total bee abundance.

For each study, we classified the origin status of honeybees as either native (in Europe, Africa and the Western part of Asia) or non‐native (in the rest of the World). Using the abundance data, we calculated the proportions of honeybees and wild bees for each city. For studies sampled in more than one city, calculations were done per city; thus, resulting in more cities than the number of studies.

We used the software R v. 4.2.1 (R Core Team 2023) for all the analyses conducted. Initially, we used Generalized Linear Mixed Models (GLMMs) to test for effects of sampling method, origin status of honeybees, the number of sampling years, the number of study sites, the city area, and the number of habitat/urban greenspace types on the proportion of honeybees sampled, fitted using a quasibinomial family and with city as a random factor to account for the nested structure of our dataset, as some cities had several studies. However, our models had convergence problems; the random variance was close to 0, and log‐likelihood ratio tests showed no differences between models with and without random structure. For these reasons, we re‐ran the models as GLMs, fitted as well with a quasibinomial family using the R package glmmTMB (Brooks et al. 2017). We additionally performed a Tukey test for differences in the proportion of sampled honeybees within the three types of sampling methodology (i.e., active, passive, both) using the package multcomp (Hothorn et al. 2008).

Furthermore, for the 37 studies where information was available, we used the individual species abundance to plot the rank‐abundance distribution (RAD) for each study. For the different analyses, we use the term datasets, which we define as the bee community data sampled in each city or urban area within a specific study. Thus, a study can provide more than one dataset if it includes more than one city.

## 3 Results

**FIGURE 2:**
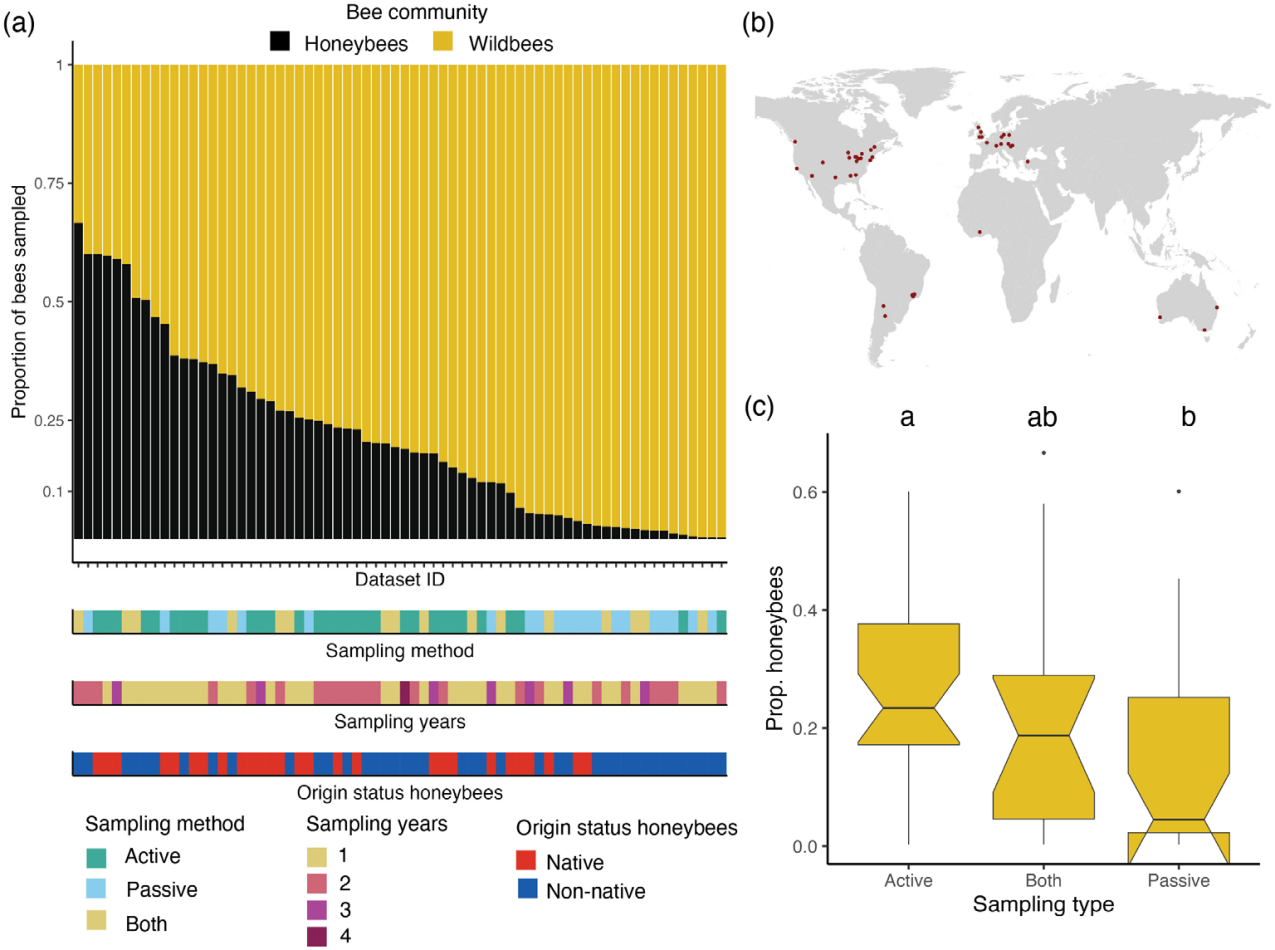
Honeybees often represent a large part of sampled urban bee faunas across cities and sampling methods. (a) Proportion of honeybees and wild bees sampled in urban areas worldwide (each bar represents a dataset, that is, an urban area linked to a study. In total, there are 68 datasets distributed among 47 urban areas). Datasets are classified according to (1) the sampling method applied for sampling bees (i.e., active = hand‐netting, observations, passive = pan‐traps, interception traps, both = active and passive methods applied together), and (2) the origin status of honeybees in the given urban areas, that is, whether honeybees are outside (non‐native) or inside (native) their natural distribution ranges. (b) Cities represented in the 68 datasets included. (c) Proportion of honeybees according to the sampling type. Letters indicate the results after performing a Tukey test for multiple comparisons to test for differences in the proportion of honeybees and the sampling type.

We obtained 58 suitable published studies from the literature review and one unpublished study. Overall, this resulted in 68 datasets representing 47 different urban areas distributed across Africa (1), Asia (1), North America (20), South America (7), Europe (15) and Oceania (3) (Figure 2). Thus, our datasets represented 17 urban areas where honeybees are native and 26 urban areas where honeybees are non‐native. We used 68 datasets (47 different urban areas) for studying the proportions between honeybees and wild bees, and 37 studies providing 41 datasets (33 different urban areas) for the RADs.

The honeybee often represented the most abundant bee species across all studies, but dominance patterns varied across the studies included. We found that honeybees accounted for 21% ± 18% (mean ± standard error) of the collected individuals (Figure 2). Specifically, in 45 of the 68 datasets, the percentage of sampled honeybees was greater than 10%. In 25 of the 68 datasets, the percentage of sampled honeybees was greater than 25%. Finally, in eight of the 68 datasets, the percentage of sampled honeybees was greater than 50% (Figure 2a). These eight datasets were predominantly from urban areas in Australia, Austria, and Argentina. The proportion of sampled honeybees was not influenced by the origin status of honeybees, the number of sampling years, the number of study sites within a city, the city area, and the number of habitat types sampled (Table S2). In fact, large percentages of collected honeybees (e.g., percentage of sampled honeybees > 10%, > 25%, > 50%) occurred regardless of the number of years sampled, the origin status of honeybees (i.e., they occurred in cities from regions where honeybees are native or exotic) (Figure 2) or the habitat type sampled (Figure S1). However, the proportion of sampled honeybees was significantly lower in studies using only passive sampling methods compared to studies using only active methods (Figure 2c and Table S2). The sampling methodology had some geographic structure at the country level, as passive sampling methods occurred especially in studies performed in the USA (10 out of 21 studies using passive methodologies were conducted in the USA).

**FIGURE 3:**
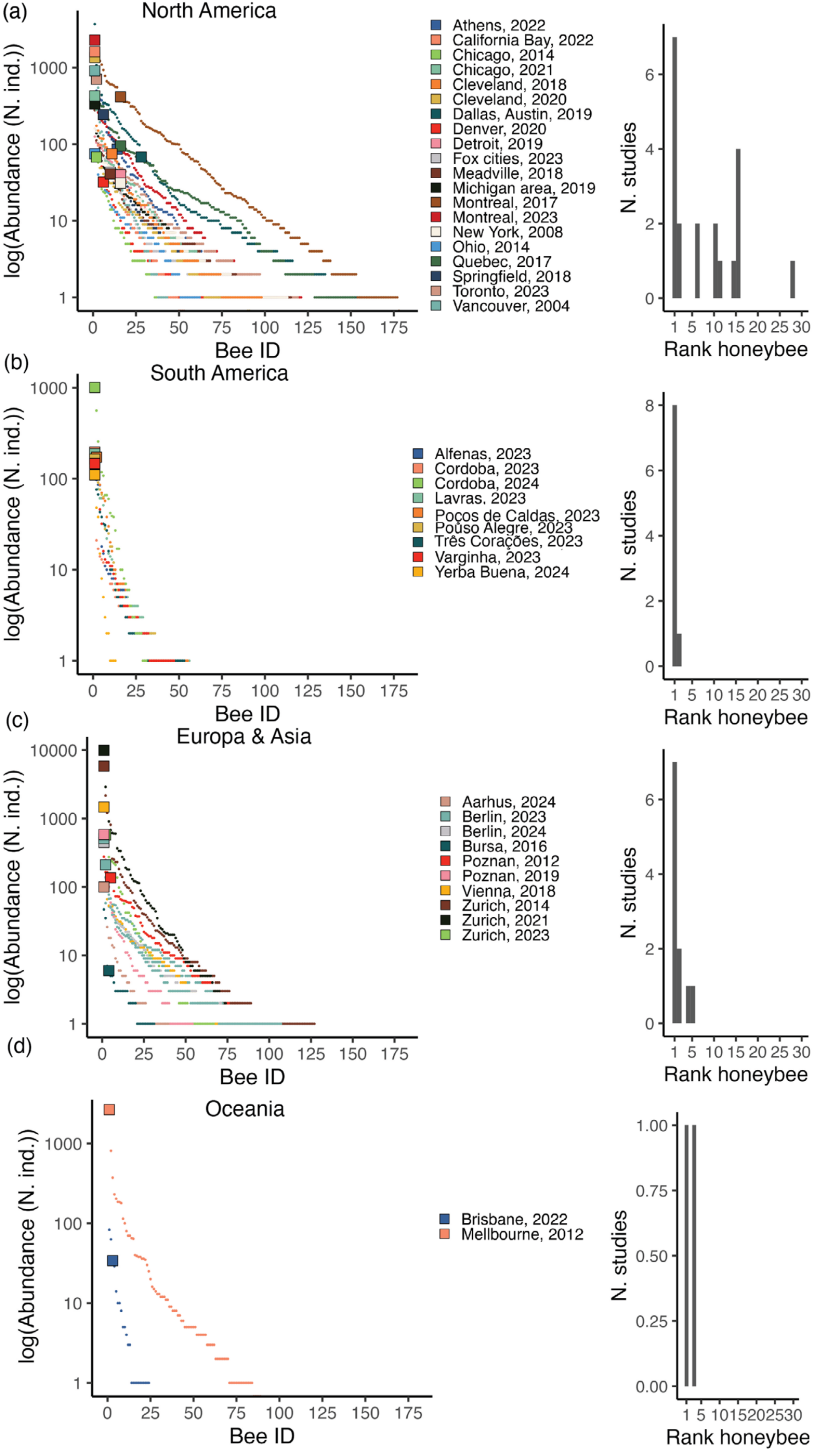
Honeybees are one of the most dominant bee species in bee faunas in cities from North America (a), South America (b), Europe and Asia (c), and Oceania (d). For each region, the rank‐abundance distribution (RAD, left plot) of sampled urban bee fauna and a histogram (right plot) of the honeybee rank in each RAD are provided. The *x*‐axis divisions represent different bee species and the *y*‐axis the number of individuals sampled. Each RAD represents a dataset, that is, an urban area linked to a study (in total, 41 datasets distributed in 33 urban areas). Hence, for some urban areas (e.g., Zurich, Switzerland) there are multiple RADs plotted representing different studies conducted in the same city. Circles represent wild bee species and larger squares represent honeybees. Note that the abundances in the RADs have been log‐transformed.

The RADs on the urban bee communities confirmed the dominance of honeybees in the bee communities for several cities (Figure 3). Specifically, we found honeybees to be among the three most abundant bee species in 29 out of 41 datasets (70% of datasets, Figure 3). Honeybees were the most abundant species in 23 out of 41 datasets (56% of datasets, Figure 3), including one out of two in Oceania, seven out of 10 in Europe, seven out of 19 in North America, and nine out of nine in South America (Figure 3).

## 4 Discussion

Honeybees are common bee species found in cities worldwide, yet quantitative estimates on their global dominance with respect to wild bees are still few. Here, we generated a comprehensive dataset reporting honeybee ( *Apis mellifera* Linnaeus 1753) and wild bee abundance in cities across 15 countries and five continents and investigated honeybee dominance patterns in these urban bee communities. Our results confirm that honeybees are dominant bee species across urban areas worldwide when considering total abundance estimates as well as the rank‐abundance distributions of bee species within the communities. However, we found that the degree of honeybee dominance varies across studies and cities worldwide. These dominance patterns agree with those reported for other ecosystem types (Herrera 2020; Lázaro et al. 2021; Wang et al. 2024).

### 4.1 Processes Increasing Urban Honeybee Populations and Dominance

Several reasons can explain the large urban honeybee populations, their abundance as a bee species in urban bee communities, and likely their high abundance in global datasets. The reasons include the growth and diversification of urban beekeeping practices, the establishment of feral populations in cities, and the spillover of foragers from the city surroundings.

The rapid growth of urban beekeeping has led to increasing honeybee hive densities in cities, driven by a mix of beekeepers, limited regulation, and lack of coordination. Honeybees are managed by humans, making their populations less affected by stressors and disturbances than wildbees. Moreover, their densities can raise quickly if the number of hives and beekeepers increases. This has been the case in several cities due to the popularity of urban beekeeping, which has led to different types of beekeepers. Particularly, urban beekeepers (e.g., individual hobbyist, commercial ventures, collectives, corporate, Egerer and Kowarik 2020; Colla 2022) differ by the number of hives managed, the intensity of beekeeping practices (e.g., number of harvests) and the purpose(s) of beekeeping (e.g., recreational, production, educational). Due to limited regulation and control by urban beekeeping associations (Sponsler and Bratman 2021) and lack of information on the current distribution and numbers of hives (e.g., Casanelles‐Abella and Moretti 2022; MacInnis et al. 2023; Matsuzawa and Kohsaka 2021, 2022), urban beekeepers are often unaware of beekeeping practices in their surrounding landscape, which might result in large densities of beehives across cities.

Honeybees can form feral colonies in cities due to swarming, a process during which a subsection of the workers with a queen departs the original hive to start a new one. The establishment of feral colonies can occur within or outside cities. For example, this process might explain the large number of honeybees reported in Cordoba, Argentina (Rotondi et al. 2023), where urban beekeeping is absent. Biotic controls by pests and parasites (such as Varroa mites) might limit the success of feral colonies, but there is evidence of feral colonies successfully established in both urban and non‐urban ecosytems (Dubaić et al. 2021; Patenković et al. 2022). Overall, the extent to which feral colonies are present and distributed in cities, as well as the processes determining their success, remains little studied.

Another process that potentially contributes to the high dominance of honeybees is the spillover of honeybee workers from hives kept in nearby agricultural areas, a phenomenon that has been documented in natural and semi‐natural ecosystems (Magrach et al. 2017; Wang et al. 2024) but less so in urban ecosystems. Spillover results in high numbers of honeybees moving to forage in the surrounding ecosystems for a certain time (Magrach et al. 2017; Wang et al. 2024). While those honeybees are transient in urban ecosystems (only present for a certain period), they can influence the structure of floral resources and intra‐ and interspecific interactions (Magrach et al. 2017; Wang et al. 2024).

### 4.2 Influence of Sampling Methodology

The methods used in a study generally did not explain high abundances of honeybees recorded. The number of study sites in a city, the city area, the number of years sampled, and the number of habitat types sampled had no influence on patterns of honeybee dominance. We could not distinguish the specific urban greenspace type where sampling was conducted. However, the large proportions of honeybees recorded across different studies set in different urban greenspace types indicate honeybees are abundant across urban greenspace types, such as private gardens (Rotondi et al. 2023), greenroofs (Braaker et al. 2014; Kratschmer et al. 2018) and community gardens and allotments (Baldock et al. 2015; Casanelles‐Abella et al. 2023; Lanner et al. 2020; Neumann et al. 2024). This suggests that dominance patterns of honeybees are generalizable across several regions and captured under different study designs and urban contexts. Nonetheless, honeybee dominance was most pronounced in studies using active sampling methods, while passive methods underestimate their presence, something that has been reported previously (Prendergast et al. 2021; Roulston et al. 2007). This discrepancy likely stems from honeybees' foraging behavior (Avarguès‐Weber et al. 2018) and learning abilities (Behrends and Scheiner 2009; Menzel 1993) that make them less attracted to pan traps compared to other pollinators (Prendergast et al. 2020). Thus, lower dominance in passive sampling studies may reflect behavioral differences rather than true community composition.

### 4.3 Varying Dominance Degree

While honeybee dominance patterns were widespread across studies and cities, the degree of dominance across cities varied. While in some cases honeybees were hyperdominant (sensu ter Steege et al. 2013) (> 50% of individuals) or highly abundant (25%–50% of the individuals), in others, their abundance ranged between 10% and 25% or even lower than 10%, making their dominance less prominent. This variability suggests city‐specific factors driving honeybee populations and their relative dominance in the urban bee community. This has ecological implications on the potential effects on bee community structure and stability, as bee dominance is expected to indicate community status (Kilpinen et al. 2022). The quantification of a critical cut‐off value for dominance that can be associated with the status of bee communities requires further research.

### 4.4 Future Prospects

Our results raise some questions regarding the implications of honeybee dominance in urban bee communities, especially outside its natural distribution, for future research. Specifically, we found that honeybee abundances can be high in cities where honeybees are within their natural range as well as in those where they are non‐native. Over the last years, there has been increasing evidence on some of the effects managed honeybees can have on wild bees, both in urban and non‐urban ecosystems and within and outside their natural distribution ranges, highlighting potential coexistence (Casanelles‐Abella et al. 2023), but also competition, disease transmission, and invasiveness (Iwasaki and Hogendoorn 2022; Sponsler and Bratman 2021). For example, previous studies have found that larger honeybee populations negatively impact overall wild bee richness (MacInnis et al. 2023) and occurrence (Herrera 2020) as well as disrupt plant visitation by other insects (Ropars et al. 2019), deplete floral resources (Torné‐Noguera et al. 2016) or enhance spillover of diseases and parasites (Geslin et al. 2017; Maurer et al. 2024). Importantly, honeybees appear to be more sensitive to changes in resource availability at larger spatial scales than wild bees. This enables honeybees to cluster or dilute within a wider landscape surrounding their hives (Casanelles‐Abella et al. 2023), whereas wild bees appear to be more restricted to smaller distances from their nesting sites (Gathmann and Tscharntke 2002). Finally, trait‐based approaches have shown that polylectic and cleptoparasitic wild bee species are negatively associated with larger honeybee populations (Lanner et al. 2025; MacKell et al. 2023; Casanelles‐Abella et al. 2023). Future studies should continue to combine species inventories with their functional traits to understand the mechanisms driving coexistence and competition between wild and managed bees.

Nevertheless, relative dominance measured at the city level is not per se a direct metric of coexistence nor competition. There are three factors that need to be further considered, including (i) urban greenspace type (or the urban habitat type) where bee communities were sampled, (ii) the temporal population dynamics of honeybees and wild bees in those sites, and (iii) the degree of floral resource overlap. The relative dominance of honeybees in each site or habitat is expected to be temporally dynamic; that is, being more pronounced in certain months likely related to seasonal changes in floral resource availability (Davey et al. 2024; Ropars et al. 2022) due to the phenology of for example, tree species (Koelzer et al. 2024). In certain habitats or sites, honeybee abundances might peak in a moment of the season that does not overlap with the phenology of other wild bee species. Even when honeybees and wild bees overlap spatially and temporally, their feeding niche overlaps and the structure of the floral resources (production, consumption and depletion rates, Sponsler et al. 2023) will determine the outcomes of their interactions. Hence, certain floral resource structures (e.g., regarding the diversity distribution of plant species, nutrients and floral trait landscapes) might facilitate niche partitioning and coexistence, whereas other structures might enhance overlap and potential competition (Sponsler et al. 2023). Lastly, it is important to note that our focus here is limited to the impact of honeybees on wild bees; however, the effects of honeybee dominance are likely influencing other pollinating insects, which can account for a large part of the pollinator communities (Baldock et al. 2019). The specific outcomes of honeybee dominance on wild bees and the full pollinator community will likely be moderated by habitat patch characteristics in urban areas, by resource availability and bee population dynamics at multiple spatial and temporal scales, but this requires further research.

## 5 Conclusions

We found honeybees to be dominant across bee communities in multiple cities, with the degree of dominance varying in magnitude. The outcomes of honeybee dominance, particularly regarding the coexistence with other pollinating animals, are still uncertain (Sponsler and Bratman 2021). However, as Sponsler and Bratman (2021) indicate, “*competition need not be constant or even frequent to be influential, since one lean year in ten might be sufficient to drive local extinctions that would persist unless reversed by immigration*”. The existence of these lag‐responses combined with the inherent complexity of biotic interactions (i.e., coexistence and competition) might limit our ability to detect changes in wild bee communities and populations soon enough to act to protect them in urban landscapes. Consequently, it is necessary to investigate the effects of honeybee dominance to then develop regulations and limitations on both managed and feral honeybee populations when and where appropriate. Such solutions should be context‐dependent (as proposed in Beaurepaire et al. 2025) considering each city specific situation. To better consider the context, it is necessary to better measure both urban bee and floral diversity and distribution. To do so, adapting existing pollinator (Potts et al. 2024) and floral resources (Michelot‐Antalik et al. 2025) sampling protocols to cities and exploring new tools (e.g., citizen science, Weissmann et al. 2023) to monitor them in space and time is thus an urgent need for municipalities that aim at safeguarding urban pollinators.

## Author Contributions

**Joan Casanelles‐Abella:** conceptualization (lead), data curation (equal), formal analysis (lead), investigation (equal), methodology (equal), project administration (lead), software (lead), visualization (lead), writing – original draft (lead), writing – review and editing (equal). **Julieta Badini:** resources (equal), validation (equal), writing – review and editing (supporting). **Katherine Baldock:** conceptualization (equal), methodology (equal), resources (equal), writing – original draft (equal), writing – review and editing (equal). **Ana Calviño:** resources (equal), writing – review and editing (equal). **Maria Silvina Fenoglio:** resources (equal), writing – review and editing (equal). **Sara Diana Leonhardt:** conceptualization (equal), methodology (equal), writing – review and editing (equal). **Astrid Neumann:** data curation (equal), resources (equal), writing – review and editing (equal). **Marco Moretti:** conceptualization (equal), data curation (equal), methodology (equal), resources (equal), writing – original draft (supporting), writing – review and editing (equal). **Mark Patterson:** conceptualization (equal), methodology (equal), writing – original draft (supporting), writing – review and editing (equal). **Bruno Rossi‐Rotondi:** data curation (equal), resources (equal), validation (equal), writing – review and editing (equal). **Aaron Sexton:** data curation (equal), methodology (equal), validation (equal), writing – review and editing (equal). **Karla Palmieri Tavares:** data curation (equal), resources (equal), validation (equal), writing – review and editing (equal). **Juan Pablo Torretta:** data curation (equal), resources (equal), validation (equal), writing – review and editing (equal). **Martin Videla:** resources (equal), writing – review and editing (equal). **Fernando Zamudio:** data curation (equal), resources (equal), validation (equal), writing – review and editing (equal). **Raffael Zenni:** data curation (equal), resources (equal), validation (equal), writing – review and editing (equal). **Monika Egerer:** conceptualization (equal), data curation (equal), methodology (equal), project administration (equal), resources (equal), supervision (equal), writing – original draft (equal), writing – review and editing (equal).

## Conflicts of Interest

The authors declare no conflicts of interest.

## Supporting information


**Appendix S1:** ece371979‐sup‐0001‐AppendixS1.xlsx.

**Appendix S2:** ece371979‐sup‐0002‐AppendixS2.docx.

## Data Availability

All data publicly available in the repository Envidat (www.envidat.ch, doi: https://www.doi.org/10.16904/envidat.652) and from the Supporting Information.
