# Supplementary material for: Examining Honeybee (Apis mellifera) Dominance Patterns Within Urban Bee Communities Worldwide
Source: Ecol Evol. 2025 Aug 13;15(8):e71979. doi: 10.1002/ece3.71979 (PMC12350035; doi:10.1002/ece3.71979)
Supplement: Supplementary file 2 — Appendix S2: ece371979‐sup‐0002‐AppendixS2.docx. [file ECE3-15-e71979-s001.docx]

**Supplementary material for the article “Honeybee dominance patterns across cities worldwide”**

**Text S1.** The aggregated cities are the Fox Cities, Wisconsin, USA (Anderson et al. 2023); California Bay (Monterrey, Santa Cruz and Santa Clara), California, USA (Quitsberg et al. 2016; Plascencia and Philpott, 2017; Cohen et al. 2021); Dallas and Austin, Texas, USA (Ballare et al. 2019) and Budapest and Vszprem, Hungary (Sülle et al. 2023).

**Table S1. Classification of the habitat/urban greenspace types sampled**. Habitat type categories are adapted from Aronson et al. 2017.

| **Habitat type category** | **Description** |
| --- | --- |
| Park and similar | Includes parks, green areas in residential estates, cemeteries and churchyards |
| Agriculture | Includes urban farmlands, alllotments, community gardens and urban agricultural sites |
| Private garden | Includes private gardens, residential yards, backyards, frontyards, home gardens. |
| Green roof | Includes green roofs. |
| Seminatural and natural vegetation | Includes vegetation types ranging from wastelands, to semi-natural to natural formations (e.g., vacant lots, reserves, grasslands) |
| Unspecified | Used when urban greenspace type is not specified. |

**Table S2.** Results of the Generalised Linear Models on the relationship between the proportion of sampled honeybees and potential drivers for the 68 datasets included.

| **Model** | **Model components** | **Estimate** | **Standard error** | **t-value** | **p-value** |
| --- | --- | --- | --- | --- | --- |
| Number of sampling years | Intercept | -1.141 | 0.316 | -3.608 | <0.001 |
|  | Sampling years | -0.088 | 0.184 | -0.479 | 0.633 |
| Number of sites | Intercept | -1.275 | 0.139 | -9.115 | <0.001 |
|  | Number of sites | 0.000 | 0.001 | -0.129 | 0.898 |
| Origin status | Intercept | -1.474 | 0.179 | -8.216 | <0.001 |
|  | Native | 0.452 | 0.265 | 1.703 | 0.093 |
| Sampling type | Intercept | -1.001 | 0.181 | -5.522 | <0.001 |
|  | Both | -0.241 | 0.324 | -0.745 | 0.459 |
|  | Passive | -0.844 | 0.337 | -2.504 | 0.015 |
| City area | Intercept | -1.211 | 0.146 | -8.281 | <0.001 |
|  | City area | 0.000 | 0.000 | -0.953 | 0.344 |
| Number of habitats | Intercept | -1.027 | 0.239 | -4.293 | <0.001 |
|  | Number of habitats | -0.152 | 0.125 | -1.222 | 0.226 |


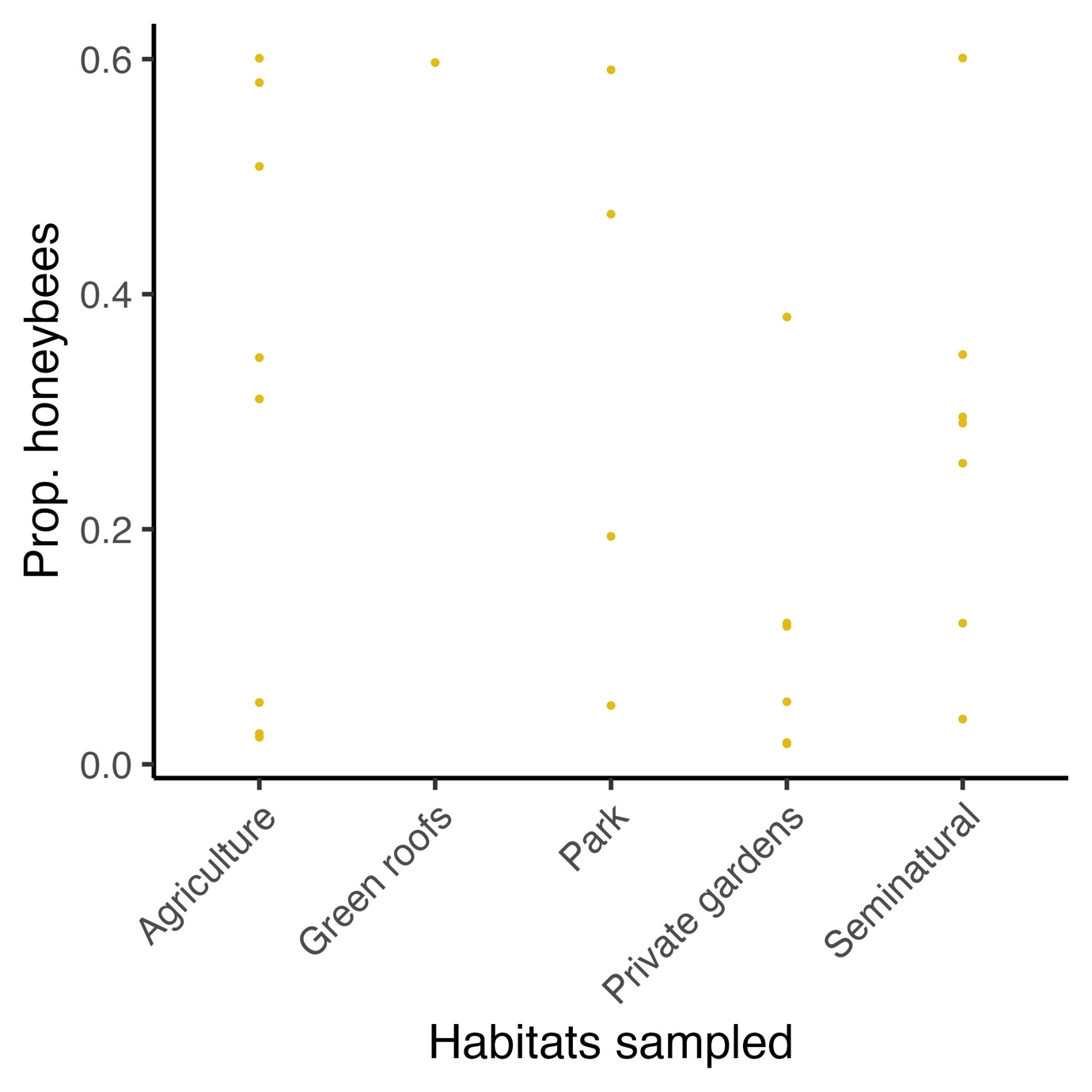


**Figure S1.** Proportion of honeybees in relation to the habitat/urban greenspace types sampled. Only datasets from studies focusing on one habitat type are plotted (26 datasets out of 68).

**References**

- Anderson, Maggie, et al. "BYO Bees: Managing wild bee biodiversity in urban greenspaces." *Plos one* 18.4 (2023): e0281468.
- Aronson, M. F., Lepczyk, C. A., Evans, K. L., Goddard, M. A., Lerman, S. B., MacIvor, J. S., ... & Vargo, T. 2017. Biodiversity in the city: key challenges for urban green space management. Frontiers in Ecology and the Environment, 15:189-196.Ballare, Kimberly M., et al. "Multi‐scalar drivers of biodiversity: local management mediates wild bee community response to regional urbanization." *Ecological applications* 29.3 (2019): e01869.
- Cohen, Hamutahl, et al. "Local and landscape features constrain the trait and taxonomic diversity of urban bees." *Landscape Ecology* (2022): 1-17.
- Plascencia, M., and S. M. Philpott. "Floral abundance, richness, and spatial distribution drive urban garden bee communities." *Bulletin of entomological research* 107.5 (2017): 658-667.
- Quistberg, Robyn D., Peter Bichier, and Stacy M. Philpott. "Landscape and local correlates of bee abundance and species richness in urban gardens." *Environmental entomology* 45.3 (2016): 592-601.
- Süle, Gabriella, et al. "First steps of pollinator-promoting interventions in Eastern European urban areas–positive outcomes, challenges, and recommendations." *Urban Ecosystems* 26.6 (2023): 1783-1797.
